# The Effect of Nintedanib in Post-COVID-19 Lung Fibrosis: An Observational Study

**DOI:** 10.1155/2022/9972846

**Published:** 2022-09-26

**Authors:** Narongkorn Saiphoklang, Pimchanok Patanayindee, Pitchayapa Ruchiwit

**Affiliations:** Division of Pulmonary and Critical Care Medicine, Department of Internal Medicine, Faculty of Medicine, Thammasat University, Bangkok, Thailand

## Abstract

**Background:**

Lung fibrosis is a sequela of COVID-19 among patients with severe pneumonia. Idiopathic pulmonary fibrosis and lung fibrosis due to COVID-19 may share many similar features. There are limited data on effects of antifibrotic treatment of infection-related lung fibrosis. This study aimed to evaluate the effect of nintedanib on patients' post-COVID-19 lung fibrosis.

**Methods:**

A retrospective, matched case-control study was performed on hospitalized patients with COVID-19 pneumonia. Patients who received nintedanib treatment for COVID-19 pulmonary fibrosis (nintedanib group) were compared to patients with standard treatment (control group). The primary outcome was oxygen improvement. The secondary outcomes were chest X-ray improvement, SpO_2_/FiO_2_ ratio improvement, mortality rates at 60 days, and adverse events.

**Results:**

A total of 42 patients with COVID-19 pneumonia were included (21 in each group). Mean age was 64.43 ± 14.59 years, and 54.8% were men. At baseline, SpO_2_/FiO_2_ ratio before treatment was 200.57 ± 105.77 in the nintedanib group and 326.90 ± 137.10 in the control group (*P* = 0.002). Oxygen improvement and chest X-ray improvement were found in 71.4% and 71.4% in the nintedanib group and in 66.7% and 66.7% in the control group (*P* = 0.739). The nintedanib group had more improvement in SpO_2_/FiO_2_ ratio than in the control group (144.38 ± 118.05 vs 55.67 ± 75.09, *P* = 0.006). The 60-day mortality rates of the nintedanib and the control groups were 38.1% vs 23.8%, *P* = 0.317. Hepatitis and loss of appetite were common adverse events (9.5% and 9.5%), while the incidence of diarrhea was 4.8%.

**Conclusions:**

Nintedanib as add-on treatment in post-COVID-19 lung fibrosis did not improve oxygenation, chest X-ray findings, or the 60-day mortality. However, this antifibrotic drug improved SpO_2_/FiO_2_ ratio in our patients. Further randomized controlled trials are needed to determine the efficacy of nintedanib for treatment of patients with post-COVID-19 lung fibrosis. *Trial Registration*. This study was registered in TCTR20220426001.

## 1. Introduction

Coronavirus disease 2019 (COVID-19) was first discovered in Wuhan, Hubei Province, China, in late 2019 [1]. The symptoms of COVID-19 infection vary, ranging from mild upper respiratory tract symptoms to severe acute respiratory distress syndrome [2]. With the development of better treatments, the COVID-19 survival rate has improved. However, many patients experience long-term post-COVID-19 sequelae, such as respiratory problems, decreased exercise tolerance, and lung damage [3, 4]. Pulmonary fibrosis is a consequence of COVID-19 infection and the basis for poor prognosis in COVID-19 patients [5, 6]. The mechanism of pulmonary fibrosis occurs after lung injury caused by stimuli such as viral inflammation or ventilator-induced lung injury, stimulating the function of fibroblasts via inflammatory markers such as transforming growth factor *β* and interleukin-6, leading to the accumulation of collagen and pulmonary fibrosis [5, 7–9]. The prevalence of post-COVID-19 fibrosis varies from 2% to 45%, depending on the severity of the virus [10–12]. The condition usually occurs in the third week of illness [11]. 10% of patients still have pulmonary fibrosis nine months after the illness [10]. Some are still tired after exertion and need oxygen support, limiting their quality of life [10, 13]. Factors associated with pulmonary fibrosis in COVID-19 patients include age, severity of disease, duration of mechanical ventilation and intensive care, smoking, and drinking alcohol [6].

Nintedanib is a tyrosine kinase inhibitor against growth factor receptors with intrinsic tyrosine kinase activity [14]. Nintedanib is an antifibrotic drug approved for the treatment of idiopathic pulmonary fibrosis (IPF) and autoimmune-related lung fibrosis, e.g., systemic sclerosis-associated ILD (SSc-ILD) [15–18]. This medication can slow the IPF progression measured by decline in forced vital capacity (FVC) [19, 20]. It has been approved for the treatment of other progressive fibrosing interstitial lung diseases (PF-ILD) [21, 22]. Interestingly, the combination of two antifibrotic drugs (nintedanib and pirfenidone) is proposed to enhance the therapeutic benefit by simultaneously acting on two different pathogenic pathways [23]. Combined nintedanib and pirfenidone treatment is superior to nintedanib monotherapy in terms of slower FVC decline in IPF [24]. There are few studies on using nintedanib as a treatment for pulmonary fibrosis caused by COVID-19 infections [25–27]. It may have a role in preventing severe lung fibrosis after SARS-CoV-2 infection, especially in patients with the PF-ILD phenotype [23].

There are limited data on nintedanib for treatment of post-COVID-19 pulmonary fibrosis. This study aimed to determine the effects of nintedanib in patients with COVID-19 lung fibrosis.

## 2. Materials and Methods

### 2.1. Study Design and Participants

We conducted a retrospective, matched case-control study with medical chart review of adult patients diagnosed with COVID-19 between January 2021 and January 2022 at Thammasat University Hospital, Thailand. We included hospitalized patients aged 18 years or older with laboratory-confirmed COVID-19 infection by polymerase chain reaction of nasopharyngeal swab or other respiratory samples. All participants had to be diagnosed with pneumonia, which was confirmed by chest radiographs. Pulmonary fibrosis was defined as the presence of reticulation, interlobular septal thickening, traction bronchiectasis, or honeycombing on a chest computed tomography (CT) scan report by a thoracic radiologist. We excluded patients with mild disease, defined as normal chest radiographs and oxygen saturation level (oxygen saturation 95% or higher).

The study was approved by the Human Research Ethics Committee of Thammasat University (Medicine), Thailand (Project No. MTU-EC-IM-0-292/64, Certificate of Approval No.004/2022), in compliance with the Declaration of Helsinki, the Belmont Report, CIOMS Guidelines, and the International Conference on Harmonization-Good Clinical Practice (ICH-GCP). Written informed consent was waived because this study was a retrospective study.

### 2.2. Procedures

Patients who received nintedanib were included in the study as the nintedanib group. Patients with standard treatments were included as the control group. Standard treatments according to local COVID-19 guidelines included antiviral agents (favipiravir or remdesivir), corticosteroids (methylprednisolone or dexamethasone), antibiotics (e.g., amoxycillin/clavulanate, ceftriaxone, and levofloxacin), anticoagulants (enoxaparin or unfractionated heparin), and immunomodulatory agents (tocilizumab or baricitinib). The nintedanib and control groups were matched by age, comorbidities, and time from symptom onset to the administration of antifibrotic agent. Nintedanib was used at 150 mg twice daily. The duration of administration depended on the physician in charge.

Clinical data including demographic characteristics, comorbidities, clinical characteristics (symptoms at initial presentation, date of pneumonia diagnosis, and CT finding), laboratory test results (complete blood count, absolute lymphocyte count, liver function test, serum creatinine, and C-reactive protein), oxygen status, oxygen saturation to fraction of inspired oxygen (SpO_2_/FiO_2_) ratio, and chest X-ray findings before and after treatment, duration of hospitalization, adverse effects of antifibrotic treatment, and status at 60-day after admission were retrieved from electronic medical records.

### 2.3. Study Outcomes

The primary endpoint was oxygen improvement, which was defined as oxygen support decreased from high-flow to low-flow oxygen devices or 3% increase of SpO_2_ detected by the same pulse oximeter. The secondary endpoints were 10% improvement in chest X-ray between before and after treatment as determined by the thoracic radiologist, adverse events, difference in SpO_2_/FiO_2_ ratio between before and after treatment, length of hospital stay, and status (alive or dead) at 60 days after admission.

### 2.4. Statistical Analysis

We hypothesized that oxygen improvement in the nintedanib and the control groups was 50% and 10%, respectively. The sample size would be 40 (20 per group) using 80% power and 5% type I error.

Data are expressed as number (%) and mean ± standard deviation. The chi-squared test was used to compare categorical variables between two groups. Student's *t*-test was used to compare continuous variables between two groups. Two-tailed *P* values of less than 0.05 were considered statistically significant. All data analyses were done on SPSS version 26.0 software (IBM Corp., Armonk, NY, USA).

## 3. Results

**Figure 1:**
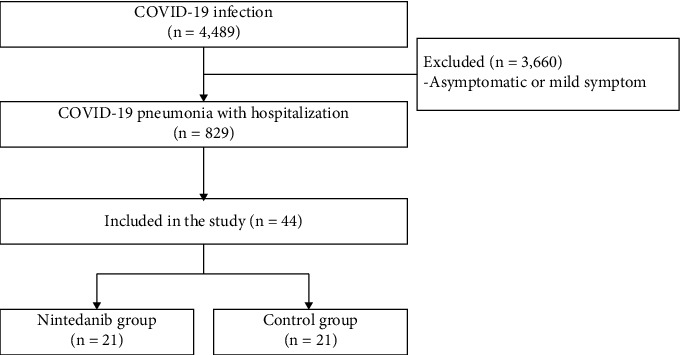
Flowchart of COVID-19 patients included in the study.

A total of 4,489 COVID-19 patients were screened. Of these, 829 patients had COVID-19 pneumonia requiring hospitalization and 44 patients (21 in each group) were included in the study (Figure 1). Mean age was 64.43 ± 14.59 years. 54.8% were male, and 11.9% were smokers. Common comorbidities included hypertension (64.3%), diabetes (50.0%), and dyslipidemia (42.9%). Cough (85.7%) was the most common symptom, followed by fever (78.6%) and breathlessness (40.5%). Baseline characteristics, laboratory results, oxygen status, and standard COVID-19 treatment were not significantly different between two groups except SpO_2_/FiO_2_ ratio and oxygen therapy before antifibrotic or standard treatment (Table 1). Mean duration of antifibrotic treatment was 17.86 ± 10.89 days (Table 2). Mean length of hospital stay in the nintedanib group was 35.48 ± 15.92 days (Table 2).

**Figure 2:**
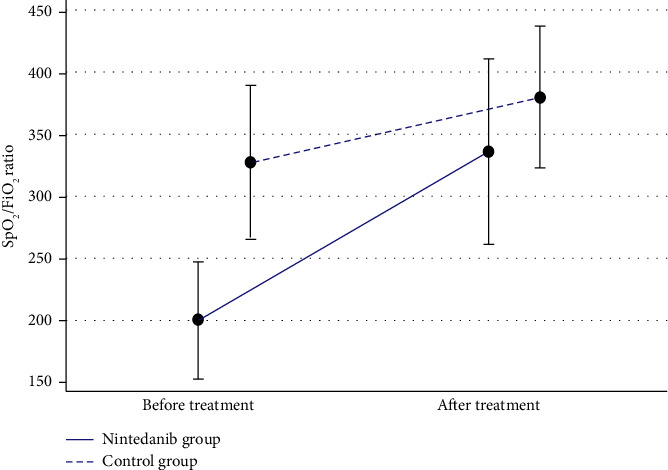
Oxygen saturation to fraction of inspired oxygen (SpO_2_/FiO_2_) difference between before and after treatment.

The nintedanib group improved significantly more in SpO_2_/FiO_2_ ratio after treatment than the control group (mean difference in 88.71, *P*=0.006) (Table 2 and Figure 2). There were no significant differences in length of hospital stay, oxygen or chest X-ray improvement, or mortality rates at 60 days after first admission between two groups (Table 2). Mortality rates at 60 days in the nintedanib group and in the control group were 38.1% and 23.8%, respectively (*P*=0.317) (Table 2).

Nintedanib side effects were hepatitis (9.5%), loss of appetite (9.5%), and diarrhea (4.8%) (Table 2). Three patients (14.3%) in the nintedanib group discontinued the medication due to hepatitis or diarrhea. No adverse effect was reported in the control group (Table 2).

## 4. Discussion

This is the first study to evaluate the effect of nintedanib in post-COVID-19 pulmonary fibrosis in Thailand. Nintedanib improved SpO_2_/FiO_2_ ratio. However, there were no differences in chest X-ray or oxygen improvement between the nintedanib and the control group. Baseline SpO_2_/FiO_2_ ratio in the nintedanib group was significantly lower than that in the control group, which may explain the greater improvement. The lack of differences in these clinical outcomes may be because of relatively short duration of antifibrotic therapy in our study (17 days). A study by Ogata and colleagues found that after 3 months of antifibrotic treatment for post-COVID-19 lung fibrosis, patients were able to reduce oxygen therapy and had better chest radiographs [25]. A study by Richeldi and coworkers found that continuing nintedanib treatment in IPF for 53 weeks showed decline in lung function, reduced relapse of the disease (acute exacerbation), and reduced the mortality rate [20].

When medication was initiated may be another important factor. Fibrosis in COVID-19 is partly caused by cytokines such as interleukin-1 and interleukin-6 during the inflammatory phase of the disease or caused by injury from mechanical ventilation that stimulates fibroblast malfunction, causing the excessive accumulation of collagen, all of which results in fibrosis [5, 7]. In our study, some patients were in acute inflammatory phase or received mechanical ventilation for treatment of severe pneumonia or acute respiratory distress syndrome, and antifibrotic drug therapy at this stage may not have been as effective as expected.

A study by Umemura and coworkers found that starting nintedanib treatment from the first day of intubation significantly reduced duration of intubation and improved chest CT findings [26].

Our study found that patients in the nintedanib group had higher 60-day mortality than the control group. Lower baseline SpO_2_/FiO_2_ ratio in the nintedanib group reflected more severe disease, which explains the higher mortality rate. Our study found that in the nintedanib group, the mean hospital stay was 35 days, similar to a previous study by Wu and coworkers [28]. Long duration of hospital stay was a risk factor for abnormal chest CT scans at 12 months after discharge from hospital [28].

Our study showed chest CT features in patients with post-COVID-19 which were consistent with previous studies (ground glass opacity, consolidation, reticulation, and traction bronchiectasis) [28–30]. A study by Liu and colleagues found that 64.7% of COVID-19 patients had normal chest CT radiographs at 4 weeks after leaving the hospital [30].

Our study found that the most common side effect of nintedanib was transaminitis, with alanine aminotransferase levels greater than 5 times greater at day 10 of treatment, requiring discontinuation of medication. Hepatitis was also found, from COVID-19 itself or from treatment-related conditions [31]. The use of antifibrotic drugs in severely ill patients with COVID-19, especially patients admitted to intensive care units, needs to be monitored more closely. Other common side effects are diarrhea and loss of appetite which are common side effects of antifibrotic drugs for treatment in IPF [20]. These side effects did not result in death in our study.

There were a few limitations of our study. Firstly, because this was a retrospective study, information was limited. It was difficult to select patient samples in the control group which had clinical characteristics and severity of disease similar to the nintedanib group. Secondly, a small sample size of the population was used in this study. Thus, study outcomes might not be representative of the whole population and some results might not be obviously different between groups. Lastly, because this was a single-center study, it might not represent the entire population. Randomized control trials with more patients are required to investigate for long-term outcomes in post-COVID-19 lung fibrosis.

## 5. Conclusions

Nintedanib in COVID-19 patients with pulmonary fibrosis did not improve oxygenation, chest X-ray findings, or 60-day mortality after admission. However, the nintedanib group had a significantly higher SpO_2_/FiO_2_ ratio difference after treatment than the control group. For future study in antifibrotic therapy for COVID-19 patients, a randomized control trial is needed to select groups of patients who would benefit from antifibrotic treatment. The appropriate timing to start medication should also be investigated for better treatment efficacy.

## Figures and Tables

**Table 1:** Baseline characteristics of hospitalized patients with COVID-19 pneumonia.

Variables	Total (*n*=42)	Nintedanib group (*n*=21)	Control group (*n*=21)	*P* value
Age, years	64.43 ± 14.59	61.29 ± 13.76	67.57 ± 15.05	0.166
Male	23 (54.8)	12 (57.1)	11 (52.4)	0.096
Body mass index, kg/m^2^	27.50 ± 5.80	26.48 ± 3.38	28.51 ± 7.45	0.189
Smoking status				
Non-smoker	37 (88.1)	17 (80.9)	20 (95.2)	0.343
Current or former smoker	5 (11.9)	4 (19.0)	1 (4.8)	0.343

Comorbidity				
Hypertension	27 (64.3)	12 (57.1)	15 (71.4)	0.334
Diabetes	21 (50.0)	10 (47.6)	11 (52.4)	0.758
Dyslipidemia	18 (42.9)	11 (52.4)	7 (33.3)	0.212
Coronary arterial disease	4 (9.5)	1 (4.8)	3 (14.3)	0.606
Stroke	2 (4.8)	1 (4.8)	1 (4.8)	1.000
Time from symptom onset to hospitalization, days	7.50 ± 14.21	5.00 ± 2.55	10.00 ± 19.86	0.259
Time from symptom onset to pneumonia diagnosis, days	7.28 ± 14.03	4.38 ± 2.48	10.19 ± 19.49	0.183

Admission ward				
Intensive care unit	18 (42.9)	10 (47.6)	8 (38.1)	0.533
Intermediate care unit	24 (57.1)	11 (52.4)	13 (61.9)	0.533

Symptom				
Fever	33 (78.6)	19 (90.5)	14 (66.7)	0.130
Cough	36 (85.7)	17 (80.9)	19 (90.5)	0.663
Breathlessness	17 (40.5)	10 (47.6)	7 (33.3)	0.346
Rhinorrhea	6 (14.3)	4 (19.0)	2 (9.5)	0.663
Sore throat	5 (11.9)	4 (19.0)	1 (4.8)	0.343
Headache	3 (7.1)	3 (14.3)	0 (0)	0.232
Anosmia	3 (7.1)	2 (9.5)	1 (4.8)	1.000
Chest tightness	1 (2.4)	1 (4.8)	0 (0)	1.000
Ageusia	1 (2.4)	1 (4.8)	0 (0)	1.000

Laboratory data				
Hemoglobin, g/dL	12.70 ± 1.79	12.85 ± 1.79	12.57 ± 1.83	0.625
White blood cell count, cells/*µ*L	7,429.27 ± 3,781.48	7,275 ± 4,097.22	7,576 ± 3,550.34	0.802
Platelet count, 10^3^/*µ*L	206.58 ± 74.98	194.00 ± 86.58	218.57 ± 61.76	0.300
Lymphocyte, %	18.62 ± 11.73	18.13 ± 12.57	19.09 ± 11.17	0.798
Absolute lymphocyte count, cells/*µ*L	1,080.12 ± 599.17	955.41 ± 443.11	1198.9 ± 707.89	0.193
Creatinine, mg/dL	1.43 ± 2.05	1.02 ± 0.53	1.82 ± 2.79	0.209
Albumin, g/dL	3.45 ± 0.48	3.47 ± 0.50	3.44 ± 0.47	0.862
C-reactive protein, mg/L	78.06 ± 55.86	83.6 ± 63.07	72.78 ± 49.02	0.542

SpO_2_/FiO_2_ ratio				
Before treatment	263.73 ± 136.79	200.57 ± 105.77	326.90 ± 137.10	0.002
After treatment	357.95 ± 148.22	335.71 ± 168.12	380.19 ± 125.44	0.338

Treatment				
Favipiravir	42 (100)	21 (100)	21 (100)	1.000
Remdesivir	5 (11.9)	3 (14.3)	2 (9.5)	1.000
Antibiotics	42 (100)	21 (100)	21 (100)	1.000
Corticosteroids	42 (100)	21 (100)	21 (100)	1.000
Anticoagulant	42 (100)	21 (100)	21 (100)	1.000
Tocilizumab	7 (16.7)	6 (28.6)	1 (4.8)	0.093
Baricitinib	2 (4.8)	1 (4.8)	1 (4.8)	1.000

Oxygen therapy before treatment				
No	10 (23.8)	2 (9.5)	8 (38.1)	0.030
Cannula	8 (19.0)	4 (19.0)	4 (19.0)	1.000
High-flow nasal cannula	8 (19.0)	8 (38.1)	0 (0)	0.003
Endotracheal tube with mechanical ventilation	15 (35.7)	7 (33.3)	8 (38.1)	0.747

Chest CT finding				
Ground glass opacity	36 (85.7)	17 (81.0)	19 (90.5)	0.663
Reticulation	29 (69.0)	14 (66.7)	15 (71.4)	0.739
Consolidation	27 (64.3)	11 (52.4)	16 (76.2)	0.107
Traction bronchiectasis	22 (52.4)	10 (47.6)	12 (57.1)	0.537
Honeycombing	2 (4.8)	2 (9.5)	0 (0)	0.488

Data shown as *n* (%) or mean ± SD. CT=chest computed tomography; SpO_2_/FiO_2_=oxygen saturation to fraction of inspired oxygen.

**Table 2:** Clinical outcomes of hospitalized patients with COVID-19 pneumonia.

Variables	Nintedanib group (*n*=21)	Control group (*n*=21)	Mean difference (95% CI)	*P* value
SpO_2_/FiO_2_ ratio difference between before and after treatment	144.38 ± 118.05	55.67 ± 75.09	88.71 (26.66 to 105.76)	0.006
Length of hospital stay, days	35.48 ± 15.92	38.57 ± 18.32	−3.10 (−13.80 to 7.61)	0.562
Oxygen improvement	15 (71.4)	14 (66.7)	NA	0.739
Chest X-ray improvement	15 (71.4)	14 (66.7)	NA	0.739
Mortality at 60 days after first admission	8 (38.1)	5 (23.8)	NA	0.317
Duration of antifibrotic treatment, days	17.86 ± 10.89	0 ± 0	17.85 (12.89 to 22.81)	<0.001

Adverse event				
Hepatitis	2 (9.5)	0 (0)	NA	0.488
Loss of appetite	2 (9.5)	0 (0)	NA	0.488
Diarrhea	1 (4.8)	0 (0)	NA	1.000

Data shown as *n* (%) or mean ± SD. NA=not applicable; SpO_2_/FiO_2_=oxygen saturation to fraction of inspired oxygen.

## Data Availability

The data supporting the results of this study are available within the article.
